# The Prevalence of Imposter Syndrome and Its Association with Psychological Distress: A Cross-Sectional Study

**DOI:** 10.3390/bs15070986

**Published:** 2025-07-21

**Authors:** Abdullah Al Lawati, Azzan Al-Wahshi, Tamadhir Al-Mahrouqi, Younis Al-Mufargi, Salman Al Shukaily, Hamood Al Aufi, Ismail Al-Shehhi, Alazhar Al Azri, Hamed Al-Sinawi

**Affiliations:** 1 College of Medicine and Health Sciences, Sultan Qaboos University, P.O. Box 50, Muscat 123, Oman; s131482@student.squ.edu.om (A.A.L.); alshukailysalman@gmail.com (S.A.S.); s133320@student.squ.edu.om (H.A.A.); shi7yyy@gmail.com (I.A.-S.); 2 Department of Behavioral Medicine, College of Medicine and Health Sciences, Sultan Qaboos University, Sultan Qaboos University Hospital, P.O. Box 50, Muscat 123, Oman; t.almahrouqi@squ.edu.om (T.A.-M.); senawi@squ.edu.om (H.A.-S.); 3 Department of General Surgery, Medical City Hospital for Military and Security Services, P.O. Box 721, Muscat 111, Oman; hashyounis96@gmail.com; 4 Sultan Qaboos University Hospital, Al-Khoudh 123, P.O. Box 50, Muscat 123, Oman; alazharalazri97@gmail.com

**Keywords:** imposter syndrome, psychological distress, depression, anxiety, students, Oman

## Abstract

This research aims to establish the prevalence of imposter syndrome among Sultan Qaboos University (SQU) undergraduate students while assessing its association with depression symptoms and anxiety symptoms. A cross-sectional design recruited 504 undergraduate students selected through stratified random sampling. Data collection employed the Clance Imposter Phenomenon Scale (CIPS), the Patient Health Questionnaire-9 (PHQ-9), and the Generalized Anxiety Disorder-7 (GAD-7). Data analysis included Pearson’s correlation, chi-square tests, and logistic regression analyses. In total, 56% of participants had imposter syndrome. The CIPS scores showed a moderate relationship with depression (r = 0.486, *p* < 0.001) and anxiety (r = 0.472, *p* < 0.001). Students who experienced imposter syndrome showed a higher probability of developing depressive symptoms (χ^2^ = 45.63, *p* < 0.001, OR = 3.49) and anxiety symptoms (χ^2^ = 32.96, *p* < 0.001, OR = 2.86). The logistic regression analysis showed that depression (B = 0.096, *p* < 0.001) and anxiety (B = 0.075, *p* = 0.003) acted as significant predictors for imposter syndrome. This study reveals a strong link between imposterism, depression, and anxiety among students. This highlights the need for university counseling programs to address imposter feelings and the role of clinical psychology in managing this phenomenon in academic and clinical settings.

## 1. Introduction

Imposter syndrome (IS) is characterized by persistent self-doubt and feelings of intellectual fraudulence despite objective evidence of success, particularly among high-achieving individuals. In high-performance sectors like medicine, academia, and competitive businesses, people often credit outside circumstances instead of their ability (Huecker et al., 2025; Langford & Clance, 1993; Clance, 1985). Those affected by IS often experience symptoms of anxiety and depression, along with an overwhelming fear of being exposed as unworthy or underqualified by their peers and colleagues (Huecker et al., 2025; Langford & Clance, 1993).

Initially introduced in 1978 by psychologists Suzanne Imes, Ph.D., and Pauline Rose Clance, Ph.D., IS was first identified in highly successful women. At that time, societal expectations, household structures, and cultural norms were believed to contribute to developing imposter feelings (Clance & Imes, 1978; Clance, 1985). However, contemporary research has shown that IS affects individuals across genders, races, and professional fields, highlighting its broad impact on personal and professional development (Bravata et al., 2020).

### 1.1. Prevalence and Importance of Addressing IS

IS is highly prevalent, with estimates ranging from 9% to 82%, depending on population and measurement tools (Bravata et al., 2020). Students, healthcare professionals, and individuals in competitive fields are especially susceptible to IS. Studies have shown that institutional biases and social pressures may cause minority and marginalized groups to experience IS at disproportionately higher rates (Cokley et al., 2013).

Dealing with IS is crucial because of its strong association with detrimental psychological repercussions. Emotional exhaustion, heightened anxiety, and ongoing stress are common symptoms of IS, and they can impair a person’s performance in both their personal and professional lives (Bravata et al., 2020). Studies have repeatedly connected poor self-confidence, more perfectionism, and a tendency to undervalue one’s achievements with imposter emotions, therefore aggravating mental health issues (Bravata et al., 2020). Understanding its prevalence in these populations can guide targeted counseling interventions to mitigate long-term psychological distress.

### 1.2. Relationship Between IS, Anxiety, and Depression

An increasing amount of data shows a clear relationship between mental health disorders, especially anxiety and depression, and IS. Research using standardized psychological tests like the Patient Health Questionnaire-9 (PHQ-9) and the Generalized Anxiety Disorder-7 (GAD-7) have revealed that those with high imposterism scores also often display high degrees of anxiety and depression symptoms (El-Ashry et al., 2024).

Perfectionistic tendencies, coupled with a persistent fear of failure, contribute to chronic stress that exacerbates depression and anxiety symptoms. People with IS can use maladaptive coping strategies such as overworking or procrastinating, which aggravates emotional pain and inadequacy. Understanding this relationship is critical for developing targeted interventions to support those affected by IS and mitigate its long-term consequences (Sheveleva et al., 2023). This triad of imposterism, anxiety, and depression presents a diagnostic challenge and emphasizes the importance of early identification within both university counseling centers and broader clinical psychology settings.

### 1.3. Consequences of IS

Beyond mental health, IS has terrible effects on general well-being, academic achievement, and career growth. In educational settings, IS is associated with test anxiety, avoidance behaviors, and procrastination, which undermine academic performance and self-efficacy. Many times, struggling with self-doubt, these students may avoid seeking treatment because they believe it would reveal their alleged lack of ability (Ménard & Chittle, 2023).

In professional environments, IS is associated with decreased job satisfaction, reduced productivity, and increased burnout. High-stress careers, such as those in education and healthcare, are particularly susceptible to the adverse impacts of IS. People in these fields typically work in settings that require continual performance reviews and peer comparison (Bravata et al., 2020). These consequences call for more studies to investigate the degree of IS among students and its correlation with mental health results.

To date, this is the first study to assess imposter phenomenon among the full undergraduate student population at Sultan Qaboos University, the largest national university in Oman. Additionally, it is the first to examine its associations with depression and anxiety using validated psychological scales. These insights offer a broader understanding of imposterism and its mental health correlates in university settings.

### 1.4. Aim of the Study

This study aims to assess the prevalence and severity of IS among Sultan Qaboos University (SQU) students by using the Clance Imposter Phenomenon Scale (CIPS) and examine its associations with depression and anxiety as measured by the PHQ-9 and GAD-7. Findings may inform the development of evidence-based mental health programs tailored for university populations and contribute to understanding imposterism in clinical settings.

## 2. Materials and Methods

### 2.1. Study Design and Setting

A cross-sectional study was designed to assess the prevalence and severity of IS among undergraduate students at SQU. SQU is the largest public university in Oman, with 9 colleges, 173 academic programs (including undergraduate and postgraduate ones), 6037 staff members, and 19,365 students, of which approximately 17,156 are undergraduate students (Planning & Statistical Department, 2023). This approach was chosen to capture experiences associated with IS and their connections to symptoms of anxiety and depression.

### 2.2. Study Population

The target population included all full-time undergraduate students enrolled at SQU across various colleges during the academic year of 2023–2024. A stratified random sampling method was applied to ensure proportional representation from multiple colleges. The final sample included 504 participants, with inclusion criteria comprising (1) currently enrolled full-time undergraduate students, including those under 18, and (2) consent to participate. In the Omani educational system, some first-year undergraduates may be 17, and all participants provided informed electronic consent approved by the university ethics committee. Students were excluded if they were part-time or postgraduate students. Confidentiality was maintained by anonymizing responses and securely storing the data. Participants were informed of their right to withdraw at any stage without penalty.

### 2.3. Data Collection

Data was collected using a self-administered questionnaire comprising a sociodemographic section and three standardized tools: the Clance Imposter Phenomenon Scale (CIPS), the Patient Health Questionnaire-9 (PHQ-9), and the Generalized Anxiety Disorder-7 (GAD-7). The questionnaire was created in Google Forms and was distributed electronically through the university’s communication platforms, and data collection was conducted over four weeks from 1 to 28 February 2025. The questionnaire was available in English, as English is the primary language of teaching, and all SQU students are proficient in it. Furthermore, all the scales utilized were validated in the English language.

### 2.4. Measurement Outcomes

In addition to a sociodemographic survey, three validated tools were utilized as follows:

CIPS: This tool determines whether an individual demonstrates IS traits, and, if so, how severe they are. The tool is in English, and participants are asked to score their level of agreement with a series of statements. Using a 5-point Likert scale, responses are categorized as “very true,” “often,” “sometimes,” “rarely,” and “not at all true.” There are 20 questions on this scale and the Cronbach’s α coefficient determined from our study is 0.857, indicating a high level of consistency. Additionally, studies using this scale have found excellent validity (Clance, 1985; Holmes et al., 1993; Chrisman et al., 1995). The range of scores is 0 to 100. Values between 0 and 40 indicate few imposter feelings, values between 41 and 60 indicate occasional imposter feelings, values between 61 and 80 indicate frequently experienced imposter feelings, and severe imposter syndrome is indicated by values higher than 80. Additionally, people were categorized as imposters if they scored 62 or higher (Holmes et al., 1993; Chrisman et al., 1995; September et al., 2001).

PHQ-9: This is a self-administered screening tool for depression consisting of 9 items, with total scores ranging from 0 to 27. Each item is rated on a scale from 0 (“not at all”) to 3 (“nearly every day”). This tool demonstrated high internal consistency in our study (Cronbach’s α = 0.823) and strong validity from previous studies (Kroenke et al., 2001). The standard interpretation of total scores is as follows: 0–4 (minimal or none), 5–9 (mild), 10–14 (moderate), 15–19 (moderately severe), and 20–27 (severe depression). While traditional cutoff scores are set at 5, 10, 15, and 20 to indicate mild to severe depression, a study by Al-Ghafri et al. found that, in an Omani population, a threshold of ≥ 12 provided the best balance of sensitivity (80.6%) and specificity (94%) for identifying clinically significant depression (Al-Ghafri et al., 2014; Al-Alawi et al., 2021).

GAD-7: This self-report scale consists of 7 items designed to assess generalized anxiety, with total scores ranging from 0 to 21. Each item is rated between 0 and 3. The GAD-7 displays excellent reliability (Cronbach’s α = 0.882) and has strong convergent validity with other anxiety measures. The original validation suggested a cutoff score of ≥9 for optimal sensitivity (89%) and specificity (82%), but validations in Arabic-speaking populations recommend a threshold of ≥10 as the most effective balance for sensitivity and specificity (Al-Alawi et al., 2021; AlHadi et al., 2017).

### 2.5. Sample Size

Given that the undergraduate student population at SQU is approximately 17,156 students, the targeted sample size for this study was 376 students (Planning & Statistical Department, 2023). The sample size was calculated using the standard formula for the population mean, with a 95% confidence level and a 5% margin of error. The targeted sample size was achieved and exceeded with 504 responses.

### 2.6. Data Analysis

Data was analyzed using IBM SPSS Statistics version 28. Descriptive statistics summarized demographic variables and psychological scores. Pearson’s correlation coefficients assessed the strength of associations among CIPS, PHQ-9, and GAD-7 scores, which was appropriate for continuous variables. Chi-square tests and odds ratios determined associations between imposterism and psychological distress, allowing for the comparison of proportions across groups. Logistic regression was used to identify significant predictors of IS, supporting the objective of identifying independent associations. Established cutoff values were applied to classify significant depression (PHQ-9 ≥ 12), anxiety (GAD-7 ≥ 10), and IS (CIPS ≥ 62). The internal consistency of the scale was assessed using Cronbach’s alpha. The overall scale demonstrated excellent reliability, with a Cronbach’s alpha of 0.913 across 36 items, indicating strong internal consistency among the questionnaire items.

### 2.7. Ethical Approval

The Medical Research and Ethics Committee (MREC#2797) at the College of Medicine and Health Sciences, Sultan Qaboos University, reviewed and approved the study on 3 June 2024. This study adheres to guidelines of the Declaration of Helsinki.

## 3. Results

As shown in Table 1, this study included 504 participants with a mean age of 20.66 ± 1.83 years. The majority were female (63.9%), while 36.1% were male. Participants were from various colleges, with the College of Medicine and Health Sciences having the highest representation (31.7%). Most participants lived with family (48.0%) or in university accommodation (32.5%).

The CIPS scores ranged from 27 to 98, with a mean of 64.41 (SE = 0.573), a standard deviation (SD) of 12.857, and a variance of 165.308. The skewness and kurtosis values were 0.038 and −0.366, respectively. The PHQ-9 scores ranged from 0 to 27, with a mean of 12.57 (SE = 0.272), an SD of 6.110, a variance of 37.335, a skewness of 0.207, and a kurtosis of −0.600. The GAD-7 scores ranged from 0 to 21, with a mean of 9.99 (SE = 0.251), an SD of 5.628, and a variance of 31.680, with skewness and kurtosis values of 0.104 and −0.843, respectively. The distribution of IS, depression, and anxiety severity among the study participants is summarized in Table 2. Most participants (58.5%) experienced high to intense IS, with 45.0% frequently affected and 13.5% severely affected. Depression severity was moderate to severe in 51.2% of participants, while 52.0% reported moderate to severe anxiety.

**Figure 1:**
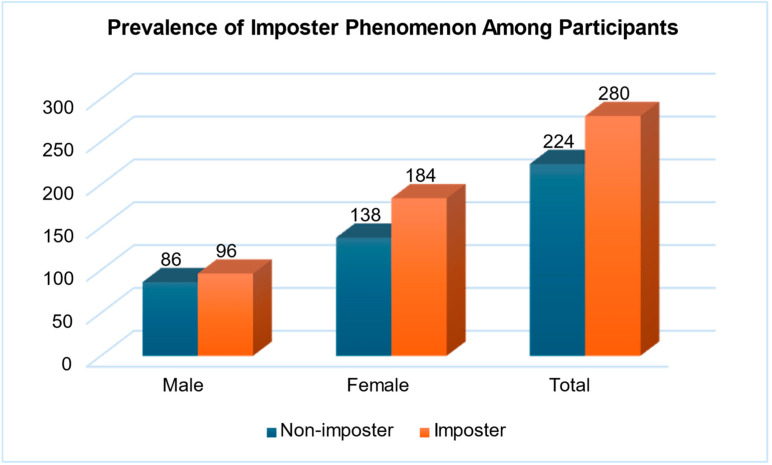
Prevalence of imposter phenomenon among participants (*n* = 504). Note: The total sample size was *n* = 504. The 95% confidence interval (CI) for the proportion of participants experiencing imposter syndrome was calculated using the standard binomial formula (95% CI: 51.67–60.33%). The cutoff score value of ≥62 differentiates imposter from non-imposter.

As illustrated in Figure 1, 56% of participants exhibited the presence of IS (95% CI: 51.67–60.33%), while 44% exhibited its absence (95% CI: 39.67–48.33%). There is no statistically significant difference in the prevalence of IP among male and female students (χ^2^(1) = 0.910, *p* = 0.340; Cramer’s V = 0.042).

As shown in Table 3, CIPS scores were moderately correlated with both PHQ-9 (r = 0.486, *p* < 0.001) and GAD-7 (r = 0.472, *p* < 0.001), indicating a significant association between IS, depression, and anxiety. A strong correlation was observed between PHQ-9 and GAD-7 scores (r = 0.735, *p* < 0.001), suggesting a close relationship between depression and anxiety symptoms.

As shown in Table 4, IS was significantly associated with both depression (χ^2^ = 45.63, df = 1, *p* < 0.001) and anxiety (χ^2^ = 32.96, df = 1, *p* < 0.001). Among the study participants with the presence of IS, 69.0% (*n* = 193) experienced significant depressive symptoms, compared to 38.8% (*n* = 87) of participants with the absence of IS. Similarly, 63.2% (*n* = 177) of participants with the presence of IS reported significant anxiety symptoms, compared to 37.5% (*n* = 84) of participants with the absence of IS.

The logistic regression analysis presented in Table 5 showed that higher depression (PHQ-9) and anxiety (GAD-7) scores significantly predicted IS. Each unit increase in the PHQ-9 score was associated with a 10% increase in the odds of experiencing IS (B = 0.096, *p* < 0.001, OR = 1.10, 95% CI: 1.05–1.15). Similarly, higher GAD-7 scores increased the odds by 7.8% per unit increase (B = 0.075, *p* = 0.003, OR = 1.08, 95% CI: 1.03–1.13). In contrast, age (B = −0.026, *p* = 0.632, OR = 0.97, 95% CI: 0.87–1.08) and gender (B = 0.092, *p* = 0.657, OR = 1.10, 95% CI: 0.73–1.64) were not significant predictors.

## 4. Discussion

### 4.1. Prevalence and Mental Health Correlates

This study highlights a concerning prevalence of IS among undergraduate students at Sultan Qaboos University, with over half of the sample meeting the criteria for clinically significant levels. These findings are consistent with international literature reporting high imposterism in academic settings, particularly among high-achieving cohorts (Bravata et al., 2020). Moreover, imposterism in our sample was strongly associated with symptoms of anxiety and depression, findings which are consistent with studies conducted among Thai students (Shinawatra et al., 2023). Students with high CIPS scores were nearly three times more likely to report moderate to severe mental health symptoms. This pattern aligns with recent evidence showing that imposterism significantly correlates with underlying psychopathology conditions, including emotional exhaustion and negative affect (Neureiter & Traut-Mattausch, 2016).

These results also resonate with findings from comparable regional contexts. A study of Saudi medical students reported that over 40% experienced imposter traits (Alrayyes et al., 2020). Internationally, similar prevalence figures have been reported with strong associations between imposterism and burnout, particularly in high-stress professions like healthcare and academia (Villwock et al., 2016; Neureiter & Traut-Mattausch, 2016). That imposterism presents similarly across regions suggests that it may be more related to academic structure and performance pressure than cultural background. At SQU, students often transition from being top achievers in secondary school to entering a highly competitive educational environment—a shift that can intensify self-doubt and reinforce feelings of imposterism and doubtfulness. Rather than any actual lack of ability, this environmental shift seems to play a central role in shaping these experiences.

### 4.2. Contextual Factors and Clinical Implications

The clinical implications are significant. Students with strong imposter feelings often delay seeking support due to fear of stigma or judgment. In cultural contexts where vulnerability is stigmatized, this may result in prolonged psychological distress and undetected academic impairment (Thompson et al., 2000). This behavior can lead to academic underperformance, reinforcing a cycle of self-doubt. In cultural contexts where vulnerability is stigmatized, such as Oman, students may be less inclined to discuss these struggles with faculty or peers (Al Lawati et al., 2023; Al Lawati et al., 2025). This has also been observed among the Eastern Asian population, where it was shown that professional expectations and hierarchical pressures led to imposterism (Choi & Lee, 2025). Since people with high imposter syndrome have been demonstrated to be reluctant to ask for assistance, especially in academic settings, this hesitancy to do so may also be a result of a fear of being perceived as insufficient (Chen & Son, 2024). The findings from this study confirm that such avoidance behaviors are not abstract but actively influence student well-being within the SQU context. Participants with elevated imposter scores were notably less inclined to seek help, despite coexisting symptoms of psychological distress. This may be further influenced by Omani cultural norms emphasizing collectivism and academic achievement as a reflection of family honor (Al Lawati et al., 2023). In such settings, fearing failure or appearing vulnerable may carry social consequences, reinforcing avoidance behaviors and internalized self-doubt. Consequently, symptoms may remain unrecognized until academic or psychological deterioration occurs.

These results suggest that academic institutions in similar cultural contexts should integrate screening for imposter traits into student mental health programs. Identifying students early—particularly those exhibiting coexisting symptoms of depression or anxiety—may enable timely and targeted intervention, reducing long-term academic and emotional consequences.

### 4.3. Intervention Strategies and Theoretical Insights

Evidence-based strategies offer a path forward. Psychoeducational workshops that normalize imposter feelings and teach attribution reframing have demonstrated promising results in student populations (Parkman, 2016). Integrating these strategies into the first-year foundation curriculum or personal development courses could be an early protective measure. Similarly, peer mentoring programs may help break the cycle of imposterism by creating supportive spaces where students can openly share experiences and navigate self-doubt together. For faculty, training programs focused on giving formative, non-judgmental feedback can help reinforce growth-oriented mindsets among students.

These efforts are particularly valuable in demanding academic fields, where the conventional attributional theory of IS highlights how individuals often misattribute their success to external factors such as luck while viewing failures as evidence of personal inadequacy (Kolligian & Sternberg, 1991). From a theoretical standpoint, these results support cognitive and attributional frameworks. Students endorsing high imposterism exhibit rigid, negative self-appraisals. Previous research shows that many attribute success to chance while interpreting minor setbacks as confirmation of incompetence (Thompson et al., 2000). These distortions, when sustained over time, can feed persistent anxiety and reduce motivation.

Our results support the real-world applicability of these models. Students with higher imposter scores were more likely to report these attributional patterns, confirming that these theoretical frameworks remain valid in the Omani academic context. Thus, interventions grounded in cognitive behavioral theory, such as structured peer support groups or reflective journaling activities, could directly challenge these thought patterns. Embedding such techniques into orientation programs may promote early resilience and reduce maladaptive attribution styles. Cognitive behavioral interventions target these thought loops by promoting more balanced interpretations of success and failure (Parkman, 2016; Gold et al., 2019; Ogunyemi et al., 2022).

These approaches may be particularly effective at SQU, where small-group, low-stigma environments could provide a culturally appropriate platform for enhancing self-efficacy and academic resilience. Theories of academic self-concept also suggest that imposter feelings are more likely to emerge in environments where positive feedback is limited or dependent on exceptional performance. Incorporating personal development modules that encourage students to recognize their efforts and abilities as the foundation of success may help counter these patterns and build greater academic resilience.

### 4.4. Strengths and Limitations

This study has notable strengths and certain limitations. Strengths include the use of stratified sampling, which ensured a diverse and representative sample across all colleges. Furthermore, the use of well-validated instruments with high internal consistency is a strength. Finally, this is the first study conducted in Oman to explore imposterism among undergraduate students and its association with anxiety and depression. As for limitations, the cross-sectional study’s nature limits our ability to establish causality, and reliance on self-reported measures may have led to bias. Nevertheless, the strong internal reliability of the measurement tools and the clear strength of the associations observed lend credibility to the findings. However, the extent to which these results can be generalized may be limited to institutions with similar academic intensity and student demographics. In addition, the higher response rate from students in the College of Medicine and Health Sciences may reflect a response interest bias, as these students could be more engaged with topics related to mental health. This may have slightly influenced the overall prevalence estimates. The predominance of female participants also reflects the broader university demographics in Oman, where female enrollment typically exceeds that of males (Issan, 2010). This national trend may partially explain the gender distribution observed in the study.

### 4.5. Future Directions

Future research should focus on longitudinal trajectories of imposterism and the effects of targeted interventions implemented in real time. Qualitative exploration may also provide valuable insights into the lived experiences of SQU students, particularly concerning how cultural values and academic expectations intertwine with feelings of imposterism. The data highlights the pressing need to integrate IS into student support frameworks. Addressing this issue is not solely about fostering individual resilience; it also reflects how institutional culture influences student identity and mental health.

## 5. Conclusions

This research shows that imposter syndrome affects many undergraduate students at SQU, and these students also experience depression and anxiety symptoms. This study shows that psychological distress is the main factor that predicts imposterism, rather than demographic factors. This study results show that universities need to develop specific mental health strategies to help students who experience imposter feelings to improve their academic success and psychological well-being. At SQU, this could involve embedding routine screening for imposter traits into student counseling or academic advisory services. Additionally, raising faculty awareness through training may help them recognize at-risk students and offer timely referrals.

## Figures and Tables

**Table 1:** Demographic characteristics of the study population (*n* = 504).

Variable	Category	N (%)/Mean ± SD
Age		20.66 ± 1.832
Gender	Male	182 (36.1)
	Female	322 (63.9)
College	College of Medicine and Health Sciences	160 (31.7)
	College of Nursing	39 (7.7)
	College of Science	58 (11.5)
	College of Law	34 (6.7)
	College of Engineering	51 (10.1)
	College of Education	40 (7.9)
	College of Economics and Political Sciences	39 (7.7)
	College of Arts and Social Sciences	53 (10.5)
	College of Agricultural and Marine Sciences	30 (6.0)
Residential status	With family at home	242 (48.0)
	With friends outside of university residence	71 (14.1)
	With friends inside of university residence	164 (32.5)
	Alone outside of university residence	27 (5.4)

**Table 2:** Distribution of imposter syndrome, depression, and anxiety in a study population (*n* = 504).

Variable Under Test	Category	N (%)
Imposter phenomenon	Few imposter feelings	17 (3.4)
	Occasional imposter feelings	192 (38.1)
	Frequently experienced imposter feelings	227 (45.0)
	Severe imposter syndrome	68 (13.5)
Depression	Minimal or none	45 (8.9)
	Mild depression	127 (25.2)
	Moderate depression	144 (28.6)
	Moderately severe depression	114 (22.6)
	Severe depression	74 (14.7)
Anxiety	Minimal anxiety	100 (19.8)
	Mild anxiety	143 (28.4)
	Moderate anxiety	139 (27.6)
	Severe anxiety	122 (24.2)

**Table 3:** Psychological correlates of imposter syndrome: associations with depression and anxiety.

Variable	r (Correlation Coefficient)	95% CI	*p*-Value	Strength
CIPS vs. PHQ-9	0.486	0.416–0.550	*p* < 0.001	Moderate
CIPS vs. GAD-7	0.472	0.401–0.537	*p* < 0.001	Moderate
PHQ-9 vs. GAD-7	0.735	0.692–0.773	*p* < 0.001	Strong

Note: Confidence intervals (CIs) for Pearson’s correlation coefficients were computed using Fisher’s Z-transformation. All correlations were tested using two-tailed significance tests, and results were considered statistically significant at *p* < 0.05.

**Table 4:** Prevalence and statistical association of imposter phenomenon with psychological distress.

IP Status	Depression		χ^2^	df	*p*-Value	OR	95% CI
	Non-Significant	Significant					
Non-imposter	137	87	45.63	1	*p* < 0.001	3.49	2.42–5.05
Imposter	87	193					
	Anxiety						
	Non-Significant	Significant					
Non-imposter	140	84	32.96	1	*p* < 0.001	2.86	1.99–4.12
Imposter	103	177					

Note: Chi-square tests were used to assess the association between imposter syndrome and psychological distress. Odds ratios (ORs) and 95% confidence intervals (CIs) were calculated by using the Mantel–Haenszel method. A PHQ-9 score of ≥12 indicates significant depressive symptoms, while a GAD-7 score of ≥10 indicates significant anxiety symptoms. IP = imposter phenomenon.

**Table 5:** Logistic regression analysis predicting imposter syndrome.

Predictor	B	S.E.	Wald	df	*p*-Value	Exp(B) (Odds Ratio)	95% CI for Exp(B) (Lower–Upper)
Age	−0.026	0.055	0.230	1	0.632	0.974	0.874–1.085
Gender (male vs. female)	0.092	0.207	0.198	1	0.657	1.096	0.731–1.644
PHQ-9 Score	0.096	0.024	15.641	1	*p* < 0.001	1.100	1.049–1.154
GAD-7 Score	0.075	0.025	8.722	1	0.003	1.078	1.026–1.133

Note: The model fit was significant, χ^2^(4) = 87.279, *p* < 0.001, with -2LL = 605.178. It explained 15.9% (Cox & Snell R^2^) to 21.3% (Nagelkerke R^2^) of the variance. The Hosmer and Lemeshow test indicated a good fit, χ^2^(8) = 9.553, *p* = 0.298, and the overall classification accuracy was 66.1%. B = unstandardized coefficient; S.E. = standard error; df = degrees of freedom; Exp(B) = odds ratio; C.I. = confidence interval; PHQ-9 = Patient Health Questionnaire-9; GAD-7 = Generalized Anxiety Disorder-7; LL = log-likelihood; R^2^ = coefficient of determination; and χ^2^ = chi-square.

## Data Availability

The datasheet may be shared upon request.
